# Guidance on selecting a translational framework for intervention development: Optimizing interventions for impact

**DOI:** 10.1017/cts.2023.546

**Published:** 2023-05-11

**Authors:** Kate Guastaferro, Angela F. Pfammatter

**Affiliations:** 1 Department of Social and Behavioral Sciences, School of Global Public Health, New York University, New York, USA; 2 College of Education, Health, and Human Sciences, University of Tennessee, Knoxville, USA

**Keywords:** Optimization, intervention development, behavior change, MOST, ORBIT, MRC

## Abstract

Intervention development frameworks offer the behavioral sciences a systematic and rigorous empirical process to guide the translation of basic science into practice in pursuit of desirable public health and clinical outcomes. The multiple frameworks that have emerged share a goal of optimization during intervention development and can increase the likelihood of arriving at an effective and disseminable intervention. Yet, the process of optimizing an intervention differs functionally and conceptually across frameworks, creating confusion and conflicting guidance on when and how to optimize. This paper seeks to facilitate the use of translational intervention development frameworks by providing a blueprint for selecting and using a framework by considering the process of optimization as conceptualized by each. First, we operationalize optimization and contextualize its role in intervention development. Next, we provide brief overviews of three translational intervention development frameworks (ORBIT, MRC, and MOST), identifying areas of overlap and divergence thereby aligning core concepts across the frameworks to improve translation. We offer considerations and concrete use cases for investigators seeking to identify and use a framework in their intervention development research. We push forward an agenda of a norm to use and specify frameworks in behavioral science to support a more rapid translational pipeline.

## Introduction

Over the past 15 years, there has been a call for a radical improvement in intervention science to accelerate the research-to-practice pipeline [1–6]. A number of approaches, methods, and/or frameworks have emerged to answer this call with the general philosophy that the behavioral interventions in which millions of dollars have been invested in are falling short [7]. In fact, it has been estimated that 86% of interventions do not make it to implementation [8]. A translational research framework for behavioral intervention development provides the necessary roadmap for a systematic approach to move from basic science to translational science. Using a translational research framework can aid in identifying the next best step in the process, thereby increasing the speed in closing the research to practice gap while simultaneously conserving precious research resources.

In what has become a sea of frameworks, it may be confusing for an investigator to know which is the best fit for a particular health problem or type of intervention. We argue that research programs need to use, specify, and report an overarching translational research framework. We focus on intervention development to disentangle the complementary but distinct processes of intervention design and implementation research as described below. We review three frameworks that exist for this purpose, clarify language-related differences between models, discuss the role of optimizing interventions as a means of increasing their utility, and provide key considerations for making a choice between frameworks. The goal is not to facilitate a battle of the frameworks nor to provide an exhaustive review, but rather to suggest a blueprint for selecting and using a framework.

## The Process of Intervention Development: Definitions

### Intervention Design vs Development

Intervention development encompasses intervention design, but, from our perspective, intervention design and development have distinct yet interrelated objectives. We operationalize intervention design to be a point within the development process during which the content, format, and delivery of the intervention are determined [9]. In contrast, we consider the intervention development process to be the systematic process of iterating and tailoring based on empirical evidence that occurs after the initial design process. This is an important distinction as there are many approaches to what we would consider intervention design (e.g., Intervention Mapping [10] and COM-B [11,12]), but that would not be considered in the larger scope of optimizing, experimenting, and evaluating for the purpose of eventual implementation.

### Optimization

Despite agreement on the need for a “comprehensive, systematic, and creative approach to revolutionizing the science of [translational research],”^4(p1)^ there is no consistent definition of optimization used in behavioral science. Wolfenden *et al*. [13] sought to identify a standardized definition of optimization in public health. Over three rounds of a modified Delphi process, a group of international public health researchers, practitioners, and policymakers defined optimization as, “*a deliberate, iterative, and data-driven process to improve a health intervention and or its implementation to meet stakeholder defined public health impacts within resource constraints*.”^13(p5)^ This definition emphasizes the *process* of optimization, applies optimization to both intervention development and implementation activities, and considers resource constraints (i.e., anything that might interfere with implementation). Missing from this definition, however, is the acknowledgment of the importance of using a principled framework to guide the deliberate, iterative, and data-driven process. Without a framework, there is the potential to optimize an intervention with unintended consequences. Just as we use experimental designs to determine the effect of our interventions and to ensure replicability and reproducibility, it is imperative we use a systematic approach to optimization to allow for the same assurances. Occasionally, optimization is conflated with the notion of adaptation. Adapting an intervention may rely on using contextual information to make an intervention better meet the needs of a context – post hoc. In contrast, optimization is about identifying and incorporating those needs into the design of the intervention from the beginning.

### Component

Behavioral interventions are nearly ubiquitously multicomponent. We operationalize a component to be any part of an intervention that can be separated out for study. A component could be intervention content, a delivery modality, or an implementation strategy. If it can be separated from the whole in a way that can be independent and manipulated, it can be a component.

## Translational Research Frameworks for Intervention Development

**Fig. 1.**
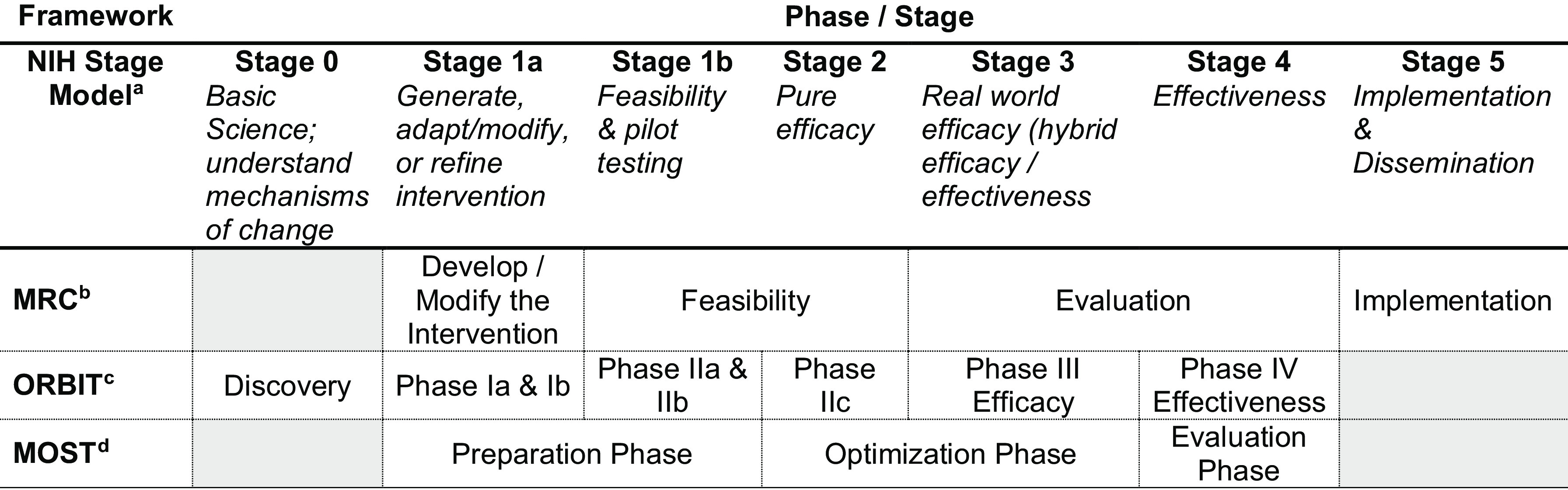
Locating the translational intervention development frameworks within the nih stage model to establish a common language MOST, multiphase optimization strategy; MRC, Medical Research Council; ORBIT, Obesity-Related Behavioral Intervention Trials. The activities of the stages for the NIH Stage Model are provided in italics so as to provide a common denominator to understand the activities and objectives of the phases specified by each of the Translational Intervention Development Frameworks. Shaded cells indicate the activities of the phases to be in the spirit of the framework, even though the particular activities are not called out in the framework. We recognize there is no definitive boundary between stages; i.e., some preparation phase work in MOST could be pure efficacy.^a14 b16 c18 d19^

For the purposes of this paper, we define translational research frameworks for intervention development to be those which span the continuum of the NIH Stage Model [14] that has a purpose of systematically developing, optimizing, and evaluating an intervention for large-scale dissemination and implementation. Three intervention development frameworks meet our definition and are the focus of this paper: the Medical Research Council (MRC) Guidelines for Complex Interventions [15,16], the National Institutes of Health’s Obesity-Related Behavioral Intervention Trials (ORBIT) model [17,18], and the Multiphase Optimization Strategy (MOST) [19]. Figure 1 labels the activities of these translational intervention development frameworks within the context of the NIH Stage Model. We provide a summary of the translational intervention development frameworks, but descriptions of each framework are not exhaustive. The reader should refer to the source materials for detailed information prior to using a framework.

## MRC Framework on Complex Interventions

The MRC guidance was initially designed to increase the use of complex interventions. By definition, complex interventions have several interacting components (including the local context) that impact the causal chain from intervention to outcome [15]. The MRC guidance suggests a flexible process of four interrelated phases: development, feasibility, evaluation, and implementation [16]. Six elements are included in all phases: (1) consider context; (2) develop, refine, and (re)test program theory (i.e., conceptual model); (3) engage stakeholders; (4) identify key uncertainties; (5) refine the intervention; and (6) economic considerations [16]. Though a phased approach, the MRC guidance does not prescribe research designs to be used in the phases. Instead, phase-specific key considerations and questions related to the complexity of components, contexts, or procedures when making methodological and practical decisions are provided. The goal is to develop an effective complex intervention that can be used in the setting for which it was designed. The guidelines adopt a systems-level approach and discuss the complexity of the system in which an intervention must operate, a critical aspect which may facilitate or hinder the public health impact [20].

MRC guidance indirectly offers suggestions of optimization, particularly as it pertains to refining an intervention to achieve goals that will enhance the possibility for implementation but does not offer an explicit definition. The MRC does not provide an iterative process or specific research designs to achieve optimization goals. Instead, it offers questions to consider during the phases that could lead one to refining the intervention or the research design to better achieve goals. To identify weaknesses in the modeling of a complex intervention such that a large-scale evaluation is unwarranted or may produce small effects [15], the guidance suggests using alternative frameworks such as MOST or RE-AIM [21].

## Obesity-Related Behavioral Intervention Trials (ORBIT) Model

Informed by the process of drug and medical intervention development, and explicitly aligned with the NIH Stage Model, the ORBIT model is used to develop behavioral interventions to prevent and/or manage chronic disease [17]. The ORBIT model was borne out of the necessity to address historical failings of behavioral intervention science. ORBIT provides a framework for behavioral researchers to engage in a phased process to provide a systematic road from basic science to translational research. A flexible, nonlinear, and iterative process as described by its proponents, ORBIT is comprised of four “purpose-guided” phases: design, preliminary testing, efficacy, and effectiveness [18]. Progression between phases is driven by the achievement of proximal behavioral and/or clinical outcomes to ensure that the intervention has a measurable effect on the chronic health outcome of interest in the end. ORBIT separates the study of the intervention from the study of an outcome.

ORBIT defines optimization as a process that can occur anywhere along the experimental phases but specifies that optimization tends to occur in Phase Ib (Refine) studies. Attempts to investigate changes to the intervention for the purpose of improving efficiency are circumscribed to the refinement phase (Phase Ib) and would not be part of later phase work. This contrasts with Phase II studies where the focus is on whether the intervention can produce a desired effect in an outcome along the intervention to clinical outcome pathway. Perhaps due to its drug development inspired etiology, the ORBIT model does not prescribe a necessary starting and/or end point for testing and development, but rather defers to the state of the science. As such, ORBIT does not specify research designs (e.g., 2-arm randomized controlled trials [RCT]) but identifies central goals and milestones (e.g., achieve an average of 5% weight loss in most participants) related to the clinical outcome of interest to be met for each phase. The overarching goal of the ORBIT framework is to provide a roadmap for a research program that follows a medical model trajectory to support the development of behavioral interventions that produce clinically significant change, improve chronic disease, and change clinical practice.

## Multiphase Optimization Strategy (MOST)

MOST is a principled framework for the development, optimization, and evaluation of multicomponent behavioral, biobehavioral, biomedical, and sociostructural interventions inspired by the fields of engineering, behavioral science, implementation science, and health economics [19]. Optimization is explicitly defined as the process of achieving intervention *EASE* by strategically balancing intervention *E*ffectiveness against *A*ffordability (i.e., the degree to which intervention is deliverable within budget), *S*calability (i.e., the degree to which the intervention is implemented in the applied setting as evaluated), and *E*fficiency (i.e., the degree to which an intervention is made up of solely active components) [22].

MOST is comprised of three phases: preparation, optimization, and evaluation [19]. In the preparation phase, the objectives are to: develop a conceptual model, identify candidate intervention components, pilot test components, and specify the optimization objective (i.e., the way in which *EASE* will be identified in this particular application of MOST). The optimization phase centers upon the empirical identification of effective components, or optimized intervention(s) that achieve intervention *EASE*. In this phase, a highly rigorous experiment is designed to understand the effect of each component individually and in combination on the outcome of interest. Empirical data are then used to determine which components meet the optimization objective and will be included in the optimized intervention. In the evaluation phase, the optimized intervention is compared to a suitable comparator usually via a randomized controlled trial.

MOST brought to the fore several innovative, rigorous experimental to empirically guide optimization (e.g., Micro Randomized Trial [MRT]; Sequential Multiple Assignment Randomized Trial [SMART] [23,24]); however, MOST does not prescribe specific experimental designs within each phase. Rather, MOST calls for the use of rigorous and efficient methods in each phase to answer essential questions posed by the science of the intervention without exceeding available or easily attainable resources. One might be guided by the state of the science to engage in a Delphi process followed by feasibility pilot trials in the preparation phase, a complex factorial (i.e., one with > 2 factors) experiment [25] or SMART in the optimization phase, and a 2-arm RCT in the evaluation phase. Because MOST offers a framework for the entire research to practice pipeline with a spirit of designing for implementation throughout, it has the potential to accelerate translational science and increase the public health impact of multicomponent interventions [26].

## Overlapping and Divergent Elements: How Do the Frameworks Compare?

As one will notice, the three translational research frameworks for intervention development share many overlapping elements but also contain conflicting and divergent elements worth discussing. All frameworks arose from the recognition that the reliance on two-arm RCTs was not serving intervention science well. Each framework acknowledges that the lack of early-stage research leads to the development and testing of intervention packages that are limited in their capacity to be disseminated out of the research context. The frameworks call on investigators to consider the need for interventions to induce a clinically (not just statistically) meaningful difference. To varying degrees, the frameworks call for more attention to mechanisms, putative targets, and adjacent outcomes important to the success and scalability (i.e., implementation and dissemination) of the intervention package to realize improvement in health outcomes outside of research. To facilitate these goals, all frameworks use a multiphasic approach to systematically develop behavioral interventions.

One of the most common points of confusion is that these translational research frameworks are not synonymous with research (or experimental) designs. Prior reviews make the mistake of classifying ORBIT as dealing with early-stage behavioral studies alone or conflating MOST with the factorial experiment. The frameworks discussed incorporate many experimental designs matched to a research question. It is best to consider the objective of each phase and properly match corresponding research questions to the experimental design that can best answer the question [27].

Frameworks diverge in their stated purpose, how steps within and between phases are prescribed, and the process of optimizing. MRC is the least prescriptive and offers a more generalized phased approach, like the NIH Stage Model. One might consider ORBIT to linearly articulate steps to take in the research pipeline. MOST uses three phases to guide the research process but recognizes and encourages working within phases until goals are met and revisiting phases to continually optimize for evolving research goals or constraints on implementation (i.e., continual optimization principle) [19]. Though all of the frameworks specify a process by which optimization can be and should be done, the phase of research in which the optimization process occurs differs. MRC uses optimization more as a key consideration without explicit guidance on the process of optimization but distinguishes intervention optimization to early phase work from the optimization of other elements, such as implementation, to later phases. MRC points to other formal structures, frameworks (i.e., MOST) and designs to direct optimization activities.

In recent descriptions of the ORBIT model, optimization is defined as “refinements necessary to move the research agenda forward based on findings from each stage [18].” Refinement is accomplished in early phase work, prior to efficacy and effectiveness trials. This is in line with the pragmatic approach espoused by the NIH Stage Model that notes the need for optimizing by removing, adding, or refining elements to support intervention implementation [14]. The result is that components or elements of an intervention might be changed based solely on a pragmatic issue such as cost, rather than driven by empirical data.

In contrast, MOST specifies that optimization occurs as an experimental process rather than a pragmatic one. Optimization is the overall goal of MOST, as evidenced by the continual optimization principle that specifies even an optimized intervention can (and should) be further optimized to consider additional or evolving science and implementation constraints. Decision-making about the composition of the intervention is done using data from the optimization phase to construct an intervention package for a new setting, context, or set of constraints. One would not make changes solely from a pragmatic point of view but instead would consider estimated effects of multiple combinations of components given certain implementation constraints to arrive at an intervention package that is more likely to produce desirable effects in evaluation. Following MOST, there seemingly is no end to a research pipeline as an intervention can be continually optimized for effectiveness, a different context, a changing reimbursement schedule, or any dissemination factor.

Beyond these overarching comparisons, there are key conceptual differences between frameworks that can guide investigators to choose the best option for their research agenda. We remind the reader the intention is not to incite a “battle of the frameworks.” The purpose is to help guide the selection of a translational research framework and improve the ability to crosswalk between frameworks as a means of improving the common language.

## MRC vs ORBIT

The primary difference between MRC and ORBIT is the degree of prescribed activities. Whereas MRC provides higher level guidance and suggestions in four main domains of development and testing, ORBIT lays out a clear roadmap of a series of phases to follow. In some ways, this means that both frameworks can be used simultaneously such that an investigator can follow the MRC guidance and consider the key issues raised while following the phasic research plan laid out by ORBIT to test and evaluate a newly developed intervention. To this end, MRC fills a key gap left unaddressed in ORBIT: the evaluation of existing interventions. It is perhaps not the intention of ORBIT, at least explicitly, to improve or optimize an existing intervention. That is not to say that an investigator could not experimentally assess the outcome of an implemented intervention in some phase of ORBIT to further develop the intervention, but MRC distinctly provides guidance on key considerations and possible research designs to guide the investigator for this purpose.

MRC lays out a cyclical process without a clear starting or end point, acknowledging the fluidity and interconnection between phases of development. On one hand, this is helpful to the individual starting with an existing intervention in an extant system that needs evaluation before further developing. While the spirit of ORBIT certainly leaves room for starting at any phase and moving between the phases, ORBIT advocates for a more linear approach, perhaps because of its origin in pharmaceutical research where the process is more linear and systems less complex. Lastly, ORBIT is heavily focused on achieving a clinically meaningful outcome whereas MRC encompasses a wide variety of outcomes of interest, not always focused squarely on a health outcome.

## ORBIT vs MOST

ORBIT is guided by medical and pharmaceutical research paradigms and provides discrete phases through which one moves once a proximal behavior change or clinical benchmark is met. It is mostly linear, though directionality is led by results of each phase and saves the RCT for last, assuming the intervention meets a priori clinical criteria for success. The research agenda could encompass other goals, but it is not explicit how to handle these other goals using the ORBIT framework. For example, early phase work might design interventions to meet the needs of a specific context from the outset. ORBIT provides little direction on what to do in the case that implementation of an intervention is no longer feasible due to changing context during the research program (e.g., cost constraints become greater over time) other than to start over in a prior phase. Thus, using ORBIT it is unclear as to how to deal with multiple components or complex interventions. Refinement and optimization are mentioned in the ORBIT framework, but a clear research methodology to guide optimization is absent. In fact, some descriptions of OBRIT refers one to MOST to optimize an intervention as if it were a research design rather than a translational research framework in and of itself.

While ORBIT would typically situate a factorial experiment testing the performance of components in a Phase Ib (Refine), we argue that when used as described by MOST, an adequately powered complex factorial trial can serve to refine as well as test the efficacy of an intervention component. For example, a factor that includes a level with the intervention component turned on and a level where it is turned off would allow the researcher to know how the component interacted with other components, but also how well it performed on its own so as to guide decisions on inclusion or exclusion of the component in future intervention packages. Thus, one might compare all those who received the component to all those who did not to know if the component was effective. When fully crossed with another component being turned on and off, a direct evaluation of the interaction is possible because the entire sample is made up of those who did not receive the components, those who received one and not the other, and those that received both, much like one might see in a 2x2 factorial (for a full review of the factorial experiment see [28]). In this example, a factorial experiment for the purpose of testing components and their interactions, when fully powered, is not well placed in Phase Ib of ORBIT. In MOST, such a trial could provide enough evidence to advance to an effectiveness trial. ORBIT, in contrast, might prescribe additional efficacy studies prior to testing an intervention package against a control in a classical randomized controlled trial. It is an important distinction in the organization of research questions and experimental methods between the two frameworks and precludes using both frameworks simultaneously or interchangeably. Furthermore, in the event the context changes, rather than reverting to prior phases, MOST would guide an investigator to first review results from an optimization trial to inform changes in the package to possibly carry forward to the evaluation phase. This example highlights one important difference in optimization goals between the frameworks. MOST has an explicit goal to use experimental designs in the optimization trial specifically to acquire data for the express purpose of making decisions about including a component in a package or sequence to be evaluated. Though this approach would not be completely inconsistent with ORBIT as it allows for flexibility in design, ORBIT falls short in providing a clear phase in which to situate research designs such as the factorial experiment or SMART because it places efficacy and refinement in different phases, not allowing for a trial design to serve multiple purposes.

There are several overlapping elements between ORBIT and MOST, particularly in the early and late phases, which one could argue are nearly identical. For this reason, it is not advisable to use both frameworks concurrently, but rather to choose between the two. For example, both frameworks call out early-phase work that occurs in Phase I in ORBIT and in the preparation phase of MOST (Figure 1). Some of the suggested research designs are the same (e.g., pilot studies), yet there are nuances around the goals of the early phases that make the frameworks different enough that a choice in framework would benefit the investigator. In ORBIT, one might look for interventions that produce a clinically meaningful difference in the health outcome whereas in MOST, one might be concerned with identifying components of an intervention that can produce a meaningful difference under a certain cost to be considered for inclusion in a treatment package. This is not to say that ORBIT requires the avoidance of resource constraints, but the consideration of resources is an explicit common thread throughout the phases of MOST in a way that it is not described in ORBIT. Finally, similar to MRC, MOST can be used to investigate a wider range of outcomes and therefore interventions, whereas ORBIT specifically focuses on behavioral interventions to improve clinical outcomes.

## MOST vs MRC

MOST is referenced in the MRC guidance as a formal framework for developing and testing complex interventions [9,16,29]. In some ways, this is accurate. MOST is a more formal and prescriptive framework compared to MRC. However, MRC also lays out phases of research that are not incompatible with the phases of MOST but may leave the researcher with conflicting advice about next steps. Much like the contrast with ORBIT, MRC places the design of an intervention in early-phase work without specifying if and how changes could be made in later phase work to accomplish goals. We argue that MOST is not a framework that can necessarily be used simultaneously with MRC, though the considerations raised by MRC may lead to the use of MOST. For example, if one seeks to evaluate an existing complex intervention that is implemented in a particular context, it would be prudent to look upon MRC guidance to provide insightful questions and ideas for going about that work. If, however, one was interested in developing a complex intervention de novo, or perhaps improving an existing intervention, MRC does not provide specific decision points for moving between phases and thus does not lay out an iterative approach to development. Instead, one might look to MOST for a principled process by which to undertake systematic development and investigation.

## Future Directions for Translational Research Frameworks

True to the scientific process, translational research frameworks will benefit from continued and incremental improvements. For example, though the MRC framework provides applied examples to underscore the “key points” to be addressed in each phase [16], there is a lack of guidance related to matching research questions to experimental designs appropriate in each phase. Over time, more specificity in and attention to methods will benefit those who choose the MRC framework. Similarly, the ORBIT model [18] would benefit from inclusion of optimization constraints (e.g., cost, time) earlier in the phases. As noted in the need to accelerate the research-to-practice pipeline and the long-standing call for optimization, it is shortsighted to not worry about cost until something is developed. An open area of research for MOST is the circumstance in which there are multiple outcomes of interest [22]. As an example, MOST would benefit from providing guidance for interventions designed to affect a clinical outcome as well as an implementation science outcome. This of course is not an exhaustive or comprehensive review of ways to improve translational research frameworks – incremental change must be responsive to the needs of those employing the frameworks.

## Key Considerations when choosing a framework

A scientist embarking on a program of research to systematically develop and evaluate an intervention to promote health by preventing or treating a chronic disease would do well to choose a guiding translational research framework, but which to choose? The answer partly depends on the type of intervention and partly depends on the goal of the research. When an intervention is complex or includes multiple components, MRC or MOST might provide clearer guidance and a more efficient path forward as compared to ORBIT. MRC provides a flexible process that researchers might find helpful for generating ideas, providing insight into appropriate methodologies to answer certain research questions, or to plan a strategy for research that will address fundamental issues to support implementation in the future. In particular, MRC could be a good starting point when an existing intervention needs evaluation and particularly when nonexperimental designs are necessary to evaluate in a certain context [30].

ORBIT presents a process by which moving through phases can guide the researcher through steps to get from discovery to practice, while prioritizing the clinical outcome. In cases where a single intervention component is a likely solution, costs are known, fixed, or minimally considered, and when the number of modifiable components is few, ORBIT is an ideal option. For example, for a single-session intervention for alcohol consumption on a college campus, ORBIT would be well suited to develop the intervention from start to finish [31]. In this example, the college campus might be less concerned with cost of the single session and are more focused on achieving the very best outcome they can and may not be interested in optimizing based on anything other than effect. In this way, the investigators can carry out the stages of ORBIT with a single goal of getting the very best effect possible. Moreover, ORBIT offers a template well known to medical colleagues, stakeholders, and entities such that acquiring funding, achieving uptake, and translating interventions could have a higher likelihood of success. For interventions with more complexities, however, an investigator might look to ORBIT and notice a paucity of guidance for prioritizing components, competing goals, or multiple outcomes. Notably, ORBIT does not provide direction on how to adjust or make decisions regarding interventions in response to a dynamic or changing context. Indeed, one could become stuck in early stages of ORBIT conducting any number of experiments over time before arriving at a feasible package to move toward later phase testing. One might also be left with little empirical information with which to make changes to an intervention once an implementation fails other than to go back to conduct additional work in the early phases.

When the goals of research are to identify interventions that balance effectiveness against efficiency, affordability, and scalability, MOST provides a systematic, principled process to efficiently develop and evaluate a multicomponent intervention. Furthermore, if it is likely (and it usually is) that the intervention may need improvement over time from an effectiveness standpoint or because contexts change, MOST espouses the continual optimization principle to guide work in a nonlinear, but systematic manner. Lastly, MOST is built in a phasic manner that enables the collection of empirical data to inform adjustments in intervention packages over time without necessarily returning to early phase work when context or constraints change. This is primarily due to explicit guidance on how to develop and empirically test components and their interactions such that the process of research includes gathering data on small pieces of an intervention. Thus, adjustments are made not from a purely pragmatic position, but informed by empirical evidence collected for the express purpose of identifying what can be added or subtracted from the intervention and the implications of doing so. While this is a notable strength of MOST, it should be noted that there is less guidance provided to the investigator in the preparation or evaluation phases as compared to the optimization phase and what is provided in the descriptions of early phases in the ORBIT model.

Up to this point we have considered choice of frameworks to be solely driven by the investigator. Yet often times funders, journal editors, or scientific team members have competing interests or a preferred framework to use (or not). In this case, it is imperative the investigator understands how to crosswalk between frameworks, particularly when research activities necessarily need to switch from one to another. Figure 1 situates each framework within the NIH stage model and also provides a template for understanding how the stages of research from each framework overlap with each other. One limitation of note is that switching between frameworks is not always advisable or possible. Early-stage feasibility work is perhaps the stage of research with the most flexibility. In feasibility studies, one might try to understand how practical an intervention component is to implement. This activity would be considered to be in Stage 1b in the NIH Stage Model, in the Feasibility Phase of the MRC, in Phase II of ORBIT, and in the Preparation Phase of MOST. The flexibility to switch between frameworks is diminished in a scenario where the investigator intends to use an optimization research design (e.g., factorial experiment, SMART, MRT). In this case, a feasibility study would also need to answer the question as to whether the component can be independently manipulated and crossed with other components. Here, switching from one framework to another, even in early phases, could lead to falling short of essential goals of one framework being met to move the work forward.

Staging becomes more complex in later stages of intervention development. As an example, a complex factorial experiment designed to test efficacy of components to screen out ineffective components in a treatment package would be in the optimization phase of MOST. In consulting the NIH Stage Model, such an activity meets goals of both Stage 1 and 2. It is unclear, based on the questions that can be answered by the complex factorial, where such an experiment is best placed in MRC or ORBIT. In this case, it is not advisable to simultaneously or interchangeably use these translational research frameworks.

## Discussion

The research to translation pipeline suffers from several issues that contribute to the lag between an intervention being developed and disseminated and ultimately affects the impact of intervention science. Some of this problem can be traced to the inefficient use of a research process without clear goals and systematic, incremental development of an intervention. The problem can also be explained by moving an intervention into effectiveness trials despite significant implementation barriers down the road. Calls to improve the pipeline are prevailing, and frameworks for intervention development have been proposed as a potential solution. Following a translational research framework can provide the investigator with a clear plan of action for a robust research program that makes compelling progress toward a meaningful improvement in public health. The proliferation of frameworks, however, creates confusion on which one to choose.

This paper is limited to frameworks that focus on the development of interventions through a translational research perspective. It does not include frameworks, approaches, or experimental research designs that are smaller in scope. For example, there are frameworks that focus on classification and/or design of an intervention and its components (e.g., Intervention Mapping [10], COM-B [11,12]). These intervention design frameworks could be used in tandem with translational research frameworks [32] but are smaller in scope and focused on the content, delivery modalities, and specifications of components of an intervention [33,34] and lack guidance on a process of evaluation. Similarly, frameworks like the Science of Behavior Change (SOBC) [17,18] may be used as an approach alongside other larger robust translational research frameworks, primarily to ensure that interventions target and perturb mechanisms as expected. There are other frameworks focused on the assessment of outcomes. Implementation frameworks (e.g., RE-AIM [21], EPIS [35], Consolidated Framework for Implementation Research [36]) provide guidance on what outcomes to measure and evaluate as part of a research program and can and should be used alongside translational research frameworks [37]. While beneficial in the translational goals of behavioral interventions, implementation frameworks fail to provide a process of systematic intervention development and, thus, are outside the scope of this paper. We argue that translational intervention development frameworks have a purpose that is specific to guiding the process of research and development. Thus, one must choose between them but can simultaneously use other frameworks that serve a different purpose. An investigator could use a translational research framework alongside an intervention design framework and an implementation evaluation framework in a way that strengthens the program of research. We argue that many design and evaluation frameworks not only complement but should absolutely be used within an overarching translational research framework.

It is not the objective of this paper to advocate for one framework over another. The purpose is to encourage the use of a framework, to inform the selection of an appropriate framework given the goal of the research program, a greater focus on optimizing to enable translation to practice, and to advocate for reporting the use of a framework in grants and manuscripts. Prior attempts to compare intervention development frameworks have suffered from broad scopes such that the approaches included encompass intervention design, research trial design, and entire translational design frameworks [38–40]. These are complementary, but not comparable. For example, a common misstep for newcomers to MOST is to describe MOST as an experimental design (e.g., “conducing a MOST trial”). This is incorrect. The optimization phase is a single phase within MOST, and there are many experimental designs suitable for the optimization trial (e.g., factorial experiment, fractional factorial experiment, SMART, MRT, etc.). Similarly, pilot studies are not a singular activity specific to the ORBIT model. Rather, pilot studies have a research objective (e.g., acceptability and feasibility) [27] included in the initial phases in all of the frameworks discussed here. Inaccurately or inconsistently distinguishing design from development as well as varying definitions of optimization has affected the impact of previous reviews of existing intervention development approaches [9,41]. Attention from both framework and methodology developers as well as intervention developers to operationally defining their conceptualization of optimization might help bring clarity to the various approaches and make a contribution to furthering intervention science.

Use of translational research frameworks with an articulated optimization process are needed to maximize the public health impact of behavioral interventions. The choice of framework should be driven by the needs of the science and the context of the clinical problem to be addressed. Regardless of choice, the use of a framework can enhance the efficiency of the research pipeline in a way that increases the likelihood that interventions move into practice. Despite the acknowledgment that a new approach is needed, there are no required reporting guidelines (e.g., intervention, randomized trial, clinical trial) that ask investigators to label and describe the framework guiding the research process. Instead, these guidelines focus on delineating the specifics of the intervention tested (e.g., mechanisms, outcomes), trial design (e.g., experimental design, comparator selection), and rationale for conducting the trial. Not specifying (and using) a framework can exacerbate the waste of research dollars on interventions that are bound to fail in implementation. There is an urgent and critical need for the inclusion of framework reporting in research checklists such as CONSORT, TIDIER, etc. We should be highly critical at this juncture of any intervention development and evaluation work that occurs without an explicit framework to guide the research process. Failing such, we will continue to see our interventions whither in the file drawer, continuing a legacy of behavioral medicine never quite making it to the table on efforts to improve health.
